# Pelvic floor muscle training delivered via telehealth to treat urinary and/or faecal incontinence after gynaecological cancer surgery: a single cohort feasibility study

**DOI:** 10.1007/s00520-023-08050-5

**Published:** 2023-09-23

**Authors:** Robyn Brennen, Sze-Ee Soh, Linda Denehy, Kuan Yin Lin, Thomas Jobling, Orla M. McNally, Simon Hyde, Jenny Kruger, Helena Frawley

**Affiliations:** 1 Department of Physiotherapy, The University of Melbourne, Parkville, VIC 3010 Australia; 2 Monash Health, Cheltenham, VIC 3192 Australia; 3 School of Primary and Allied Health Care, Monash University, Frankston, VIC 3199 Australia; 4 School of Public Health and Preventive Medicine, Monash University, Melbourne, VIC 3004 Australia; 5 School of Health Sciences, The University of Melbourne, Parkville, VIC 3010 Australia; 6 The Peter MacCallum Cancer Centre, Melbourne, VIC 3000 Australia; 7 School and Graduate Institute of Physical Therapy, National Taiwan University, Taipei City, 100 Taiwan; 8 Monash Health, Moorabbin, VIC 3189 Australia; 9 The Royal Women’s Hospital, Parkville, VIC 3010 Australia; 10 The University of Melbourne, Parkville, VIC 3010 Australia; 11 Mercy Hospital for Women, Heidelberg, VIC 3084 Australia; 12 Auckland Bioengineering Group, University of Auckland, Auckland, New Zealand

**Keywords:** Pelvic floor muscle training, Urinary incontinence, Faecal incontinence, Gynaecological cancer, Telehealth, Biofeedback

## Abstract

**Purpose:**

To assess the feasibility and clinical outcomes of telehealth-delivered pelvic floor muscle training (PFMT) for urinary incontinence (UI) and/or faecal incontinence (FI) after gynaecological cancer surgery.

**Methods:**

In this pre-post cohort clinical trial, patients with incontinence after gynaecological cancer surgery underwent a 12-week physiotherapist-supervised telehealth-delivered PFMT program. The intervention involved seven videoconference sessions with real-time feedback from an intra-vaginal biofeedback device and a daily home PFMT program. Feasibility outcomes included recruitment, retention, engagement and adherence rates. Clinical outcomes were assessed at baseline, immediately post-intervention and a 3-month post-intervention using International Consultation on Incontinence questionnaires for UI (ICIQ-UI-SF) and Bowel function (ICIQ-B) and the intra-vaginal biofeedback device. Means and 95%CIs for all time points were analysed using bootstrapping methods.

**Results:**

Of the 63 eligible patients, 39 (62%) consented to the study. Three participants did not complete baseline assessment and were not enrolled in the trial. Of the 36 participants who were enrolled, 32 (89%) received the intervention. Retention was 89% (*n*=32/36). The majority of participants (*n*=30, 94%) demonstrated high engagement, attending at least six videoconference sessions. Adherence to the daily PFMT program was moderate, with 24 participants (75%) completing five-to-seven PFMT sessions per week during the intervention. All clinical outcomes improved immediately post-intervention; however, the magnitude of these improvements was small.

**Conclusion:**

Telehealth-delivered PFMT may be feasible to treat incontinence after gynaecological cancer surgery.

**Trial registration:**

ClinicalTrials.gov Identifier: ACTRN12621000880842)

**Supplementary Information:**

The online version contains supplementary material available at 10.1007/s00520-023-08050-5.

## Introduction

Gynaecological cancers account for 16% of cancers diagnosed in women worldwide [1]. Gynaecological cancer treatments may impact bladder, bowel and pelvic floor muscle (PFM) function [2–5]. Higher rates of urinary incontinence (UI) and faecal incontinence (FI), and decreased PFM function, are found in women after gynaecological cancer treatment, compared to non-cancer populations [2, 3, 6]. There is level 1 evidence for PFM training (PFMT) to treat UI [7] and level 2 evidence for PFMT to treat FI [8]. However, due to the potential impacts of cancer treatment [2, 3], women who have had gynaecological cancer treatment may respond differently to PFMT compared to women who have not had gynaecological cancer treatment. Given that evidence from studies of PFMT in other populations cannot be directly applied to patients who have had gynaecological cancer treatment, population-specific trials to investigate PFMT to treat UI and/or FI after gynaecological cancer treatment are needed.

Telehealth is the provision of health care remotely via digital communication technology, usually through videoconference [9, 10]. During the COVID-19 pandemic, telehealth was especially important for cancer patients to avoid exposure during periods of immune compromise, such as during and after cancer treatment [11]. Providing PFMT via telehealth without an in-person assessment presents specific challenges. Correct technique is essential for successful PFMT [12]. However, the clinician is unable to assess and confirm that the patient is performing a correct PFM contraction during a telehealth consultation. Use of an intra-vaginal device, which provides real-time information about the patient’s PFM contraction technique to clinician and patient [13], provides an opportunity to assess PFM function remotely.

A 2020 systematic review found insufficient data to evaluate the impact of PFMT on UI/FI after gynaecological cancer treatment due to a limited number of trials, most of which were very small [14], and none of which included telehealth-delivered PFMT. To date, no studies have investigated telehealth-delivered PFMT to treat UI/FI after gynaecological cancer treatment without a clinical assessment. One study investigated the feasibility of telehealth-delivered PFMT to treat stress UI in women with breast cancer and found that it may be feasible and potentially beneficial [15]. However, we do not know if similar results will be observed in women with gynaecological cancer due to differences in demographics, and clinical and treatment characteristics between breast and gynaecological cancer survivors. The feasibility of recruiting to and delivering such an intervention in this population remains unknown and should be investigated prior to investing research resources in large randomised controlled trials (RCTs) of telehealth-delivered PFMT to treat UI/FI after gynaecological cancer treatment.

Our primary aim was to assess the feasibility of recruiting to and delivering a telehealth-delivered PFMT program to treat UI/FI after gynaecological cancer treatment, in order to inform the design of a larger RCT. We hypothesised that PFMT would be safe and feasible after treatment for gynaecological cancer. Our secondary aim was to assess pelvic floor clinical signs and symptoms before and after intervention.

## Methods

This study is reported according to the Standard Protocol Items: Recommendations for Interventional Trials (SPIRIT) statement [16], and the Consensus on Exercise Reporting Template (CERT)-PFMT variation [17]. Ethics approval was obtained from the Monash Health Human Research Ethics Committee (NMA HREC Reference Number: HREC/50085/MonH-2019-165068(v1)).

### Trial design and setting

This is a pre-post single group feasibility clinical trial. The trial was registered with the Australian New Zealand Clinical-Trials Registry (registration number ACTRN12621000880842).

### Eligibility criteria

We included women (≥18 years old) who had completed surgical +/− adjuvant treatment for endometrial, uterine, cervical or ovarian cancer (stages I–III) and experienced UI at least once in the preceding 4 weeks [18] or FI at least once in the preceding 3 months [19]. Women who were pregnant, breastfeeding, had neurological disorders or severe physical or psychiatric impairments, had pelvic surgery for incontinence or pelvic organ prolapse in the preceding 2 years, were within 6-week post-surgery or 3-month post-adjuvant therapy, or were unable to communicate in English were excluded. Women required a mobile device with Internet access to participate.

### Recruitment

From January 2022 to March 2022, inclusive, electronic patient lists from the gynaecological-oncology clinics at two participating tertiary hospitals in Melbourne were screened by a member of the research team to identify potential patients who may be eligible for this study. Potentially eligible patients were then screened by clinical staff against the inclusion criteria and asked to provide consent to be contacted by a research team member. The research team member contacted patients in-person or by phone to screen further inclusion and exclusion criteria, including the frequency of UI/FI symptoms, discuss study details and provide consent forms (in-person, by post or online). Participants from previous studies conducted by the researchers who had given permission for contact for further studies were also contacted and screened for eligibility using the same procedure.

Patients who consented were sent a link to the first online questionnaire by email. Patients who completed the online questionnaire were enrolled into the study and were sent a femfit® (Junofem) intra-vaginal pressure biofeedback device via post prior to the first telehealth session. Participants started the intervention between February and May 2022, inclusive. After the first session, participants had fortnightly follow-up sessions for 12 weeks. Participants completed the first post-intervention questionnaire and femfit® reassessment at 0–2 week post-intervention and the second post-intervention questionnaire at 3-month post-intervention. The final participant completed the intervention in August 2022 and completed the 3-month post-intervention follow-up in October 2022.

### Intervention

Participants underwent a 12-week telehealth-delivered PFMT program using the femfit® device. Telehealth sessions were conducted by a registered physiotherapist with postgraduate qualifications and 16 years’ clinical experience in pelvic floor physiotherapy. Each session was of 30–60 min duration. Participants joined the telehealth sessions from their homes using the Zoom^TM^ videoconference application on their mobile device. There were no in-person sessions during the assessment or intervention. Further details of the intervention can be found in the ANZCTR registration (https://www.anzctr.org.au/Trial/Registration/TrialReview.aspx?id=381893&isReview=true).

**Fig. 1:**
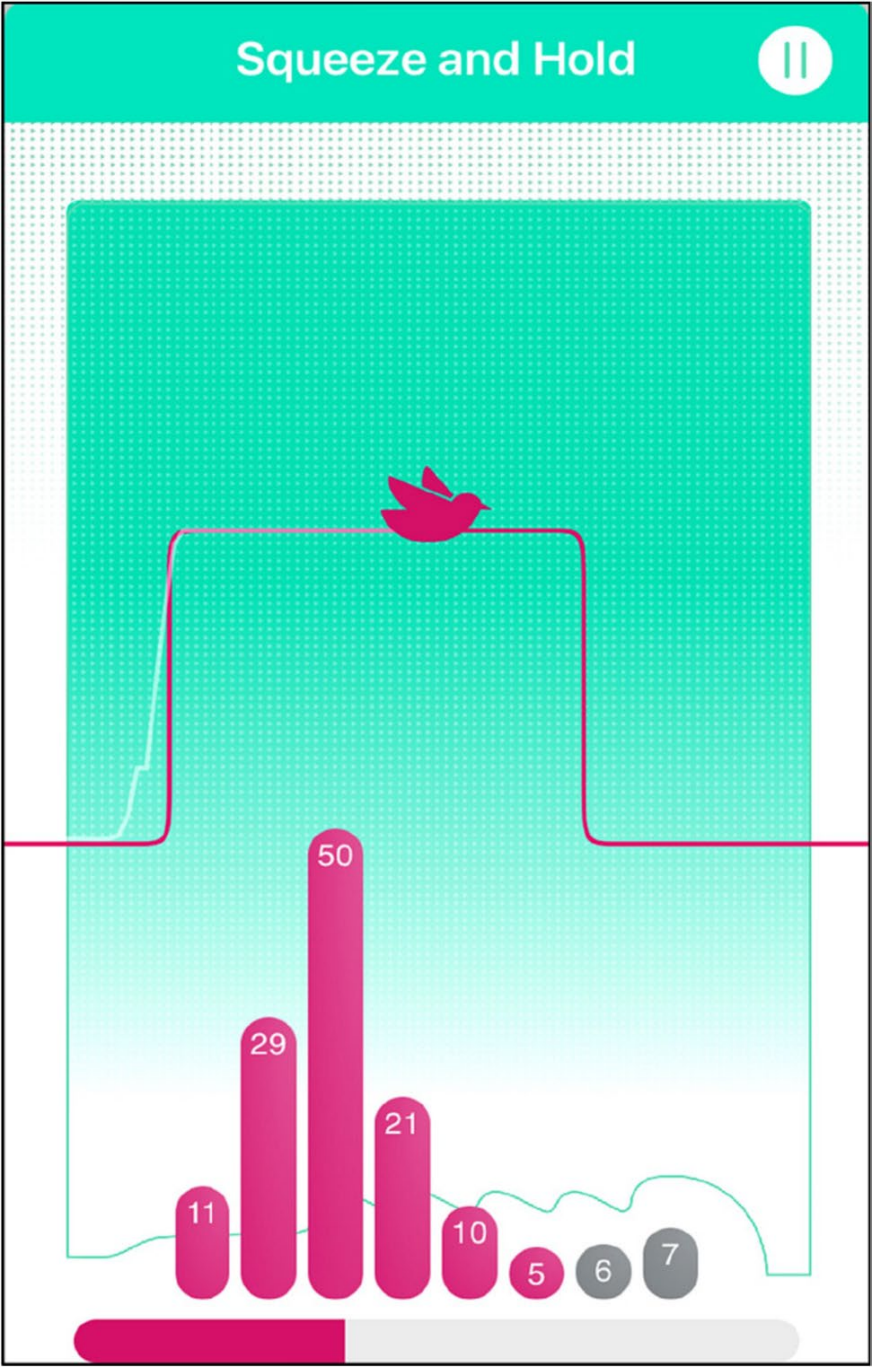
Femfit® display during a pelvic floor muscle contraction

In the first telehealth session, participants were guided through how to contract their PFMs, use the femfit® and complete their home exercise program. The pressure readings from the femfit® were displayed via an application on their mobile phone or tablet and viewed in real time by the physiotherapist using remote screen sharing. The display consisted of six pink bars representing pelvic floor pressure and two grey bars representing intra-abdominal pressure, and a bird that flew higher when pelvic floor pressure increased and lower when it decreased (Fig. 1). This provided biofeedback to the participants and was used by the physiotherapist to verbally cue correct PFM contraction technique. Correct technique which was indicated by the pink bars rising higher than the grey bars, confirmed that pelvic floor pressure was higher than intra-abdominal pressure.

Participants received seven supervised, individual PFMT telehealth sessions, during which they used the intra-vaginal sensor. They followed a pre-defined daily home exercise program installed on the femfit® mobile device application which was based on a published PFMT program [20]. The daily PFMT included three sets of six-to-ten maximal contractions, six-to-ten fast contractions, three endurance contractions and three contractions with cough (knack) during the intervention phase. Every 4 weeks, the number of repetitions and duration of each contraction increased, and positions were progressed from across-gravity (lying) to against gravity (sit, then stand). The program was tailored to each individual by delaying the progression timings if the participant was not yet able to achieve the scheduled progression. Education-based therapies were also provided alongside PFMT depending on the participant’s symptoms. These included PFM pre-contraction for increases in intra-abdominal pressure, fluid management, urgency suppression techniques, urge trigger desensitisation strategies, voiding and defecation dynamics, toilet posture and dietary fibre intake [21]. After completing the 3-month intervention, participants were encouraged to continue using the femfit® sensor and application without supervision, with a maintenance program 3 days per week of three sets of 12 maximal contraction, three sets of 12 fast contractions, three endurance contractions and three knack contractions.

Participant’s adherence to the exercise program was monitored using an exercise diary incorporated into the femfit® phone application, which also acted as a motivational strategy. Other motivational strategies included information on benefits of PFMT, action planning, process goal setting, exploring barriers and enablers to completing exercises and setting reminder notifications for the home exercise program [22].

### Outcomes

#### Sociodemographic and medical outcomes

Sociodemographic and medical outcomes including age, height, weight, parity, home situation, relationship status, education level, employment status, smoking status, medical history and cancer history were collected via online questionnaire prior to starting the intervention.

#### Feasibility outcomes

Feasibility outcomes were based on National Institute for Health and Care Research guidelines [23] and the Eldridge et al. [24] framework for describing pilot and feasibility studies. These were collected during recruitment, during intervention and at the 3-month follow-up timepoint.

- Coverage was calculated by the proportion of women who were identified as eligible for the study, who then consented.
- Retention rate was calculated at the end of the follow-up period using the number of participants who provided follow-up data at the 3-month post-intervention time point.
- Engagement was measured by the attendance rate, which was calculated at the end of the intervention period using the number of telehealth sessions attended out of seven.
- Adherence to the home exercise program during the intervention period was calculated at the end of the intervention period using the average number of days per week that the participant completed the home exercise program, as recorded in the femfit® exercise diary. Completion of exercise sessions was automatically recorded in the femfit ® application. Exercise sets could also be added manually by the participant if they completed the exercise without using the femfit® application. Adherence to ongoing PFMT during the follow-up period was assessed by self-report questions on how many times per week the participant had completed PFMT.
- Fidelity of treatment receipt [25] for the home exercise program was assessed in each telehealth session by participants’ ability to verbally describe the home program components and a screenshot of their femfit® exercise diary, documented in session notes by the researcher.
- Acceptability of the trial protocol to clinicians was assessed by the proportion of potentially eligible participants referred to discuss the trial with the research team. Acceptability of the study protocol to participants was assessed by study-specific questions in the final follow-up questionnaire. Acceptability of the different components of the program was assessed using a seven-point numerical rating scale anchored at 1=very unacceptable and 7=very acceptable.
- Satisfaction with the intervention was assessed using a seven-point numerical rating scale anchored at 1 (very dissatisfied) and 7 (very satisfied).

#### Clinical outcomes

The clinical outcomes were measured at baseline, 0–2 weeks post-intervention and at the 3-month follow-up time point:

- Prevalence, frequency, severity and impact of UI were assessed using the International Consultation on Incontinence Questionnaire-Urinary Incontinence Short Form (ICIQ-UI SF) [18]. The ICIQ-UI SF is a self-administered patient-reported outcome that has been validated for use in women with UI. It consists of items assessing frequency, severity and impact of UI in the preceding 4 weeks, the scores of which are summed to give an overall score, as well as an unscored item assessing type of UI. Scores range from 0 to 21 with higher scores indicating higher impairment of UI. The ICIQ-UI SF has been shown to have good internal consistency, test-retest reliability, and content, convergent and discriminant validity [26, 27]. The presence of UI was identified by any score >0 on the ICIQ-UI SF.
- Bowel function was assessed using the Consultation on Incontinence Questionnaire-Bowel (ICIQ-B) [19]. The ICIQ-B is a self-administered patient-reported outcome that has been validated for use in women with FI. It consists of 20 items, with three subscales assessing anorectal symptoms, bowel control and impact on quality of life associated with anal incontinence symptoms in the preceding 3 months. Subscales are scored with scores ranging from 1 to 21 for bowel pattern, 0 to 28 for bowel control and 0 to 26 for impact on quality of life, with higher scores indicating higher impairment or impact. The ICIQ-B has been shown to have good internal consistency, test-retest reliability and content and convergent validity [28, 29]. The presence of FI was assessed by any response other than ‘always’ to questions 9a and 10a on being able to control accidental loss or leaking of stool.
- Pelvic floor muscle strength and endurance were assessed using the femfit® device. Sensors 3–6, which sit at the level of the levator ani muscle, were used to assess PFM pressure [30]. It was likely that some participants would fatigue after the first few contractions [31]; however, others could experience a learning effect resulting in later contractions being stronger [32]. We therefore used the mean of the three highest PFM pressures from any of the maximal contractions within the exercise program to calculate the PFM strength score in millimetres of mercury (mmHg). For the endurance, the average PFM pressure of each of three 18-s endurance contractions was calculated, and the highest of these was used as the PFM endurance score in mmHg. The femfit® device has been shown to have excellent test-retest reliability in measuring intra-vaginal squeeze pressure during PFM contractions [13].
- Health-related quality of life was assessed using the European Organization for Research and Treatment of Cancer Quality of Life Core Questionnaire (EORTC QLQ-C30). The EORTC QLQ-C30 comprises 30 items assessing functional and symptom aspects of HRQoL. All scale/single item measures range in score from 0 to 100 [33]. A high score on the functional and the global QoL scales represents a high level of functioning and high quality of life, while a high score on symptom scales represents a high level of symptomatology [33]. The EORTC-QLQ C30 has been shown to have moderate-to high internal consistency, reliability and validity [33–35].

### Sample size

The primary aim for this study was to investigate feasibility, with primary feasibility outcomes of coverage, retention and engagement. Based on coverage of 25% of eligible patients experienced in previous studies of similar interventions by researchers in this team, and an estimated potentially eligible participant pool of *n*=120 over 9 months, we aimed to recruit 30 participants. Furthermore 30 participants should provide sufficient confidence to observe and estimate attrition and adherence data to inform a large RCT [36]. To allow for 15% attrition, we aimed to recruit 35 participants.

### Statistical methods

Descriptive statistics were reported for participant demographics, summary scores from questionnaires and feasibility data. Binary clinical outcome data were reported as proportions with 95% confidence intervals (95%CIs) using the Agresti-Coull method [37]. Continuous clinical outcome data were visually assessed for normality. Since the majority of these outcomes were skewed, means and 95%CIs were calculated using the bootstrap method. Comparisons between pre- and post-intervention measures were made using bootstrap paired *t* tests for continuous outcomes and generalised estimating equations for binary outcomes. As this was a feasibility study, the results were reported as 95%CIs, without *p* values. All analyses were conducted by RB under the guidance of a statistician (CP) using IBM SPSS Statistics (Version 27).

## Results

### Participant characteristics

We recruited 36 participants with demographic and clinical characteristics shown in Table 1. Over-recruitment occurred due to a delay between participants being sent and then completing consent forms.

**Table 1:** Participant characteristics at baseline

Characteristics	All participants (*n*=36)
*All values median (IQR)*	
Age	58 (17)
Body mass index	32.8 (13.5)
*All values n (%)*	
Living situation	
- Home alone, independent - Home with family - Home with supports - Retirement village - Other	6 (16.7) 28 (77.8) 0 (0) 0 (0) 2 (5.6)
Employment status	
- Working full-time - Working part-time or casual - Sick leave - Not employed - Retired - Home duties - Studying	14 (38.9) 8 (22.2) 0 (0) 1 (2.8) 9 (25.0) 3 (8.3) 1 (2.8)
Highest level of education	
- Completed primary school - Completed high school - Completed trade, community, TAFE, college - Completed undergraduate degree - Completed Masters or PhD - Other	0 (0) 14 (38.9) 11 (30.6) 6 (16.7) 3 (8.3) 2 (5.6)
Relationship status	
- Single - In a steady relationship - Living with partner or married - Divorced or separated - Widowed - Other	2 (5.6) 2 (5.6) 26 (72.2) 5 (13.9) 0 (0.0) 1 (2.8)
Parity - Nulliparous - Parous	7 (19.4) 29 (80.6)
Menopausal status	
- Pre-menopausal - Peri-menopausal - Post-menopausal - Unknown	6 (16.7) 1 (2.8) 28 (77.8) 1 (2.8)
Hormone replacement therapy	
- No HRT - Previous or current HRT	30 (83.3) 6 (16.7)
Characteristics	All participants (*n*=36)
Smoking status	
- Never smoked - Used to smoke - Currently smoke	22 (61.1) 11 (30.6) 3 (8.3)
Location of cancer	
- Endometrium/uterus - Endometrium/uterus + ovaries - Endometrium/uterus + cervix - Endometrium/uterus + cervix + ovaries - Cervix - Ovaries	18 (50.0) 3 (8.3) 3 (8.3) 1 (2.8) 7 (19.4) 4 (11.1)
Stage of cancer before treatment	
- Stage 1 - Stage 2 - Stage 3	24 (67) 8 (22) 4 (11)
Type of cancer treatment	
- Surgery only - Surgery + adjuvant therapy o Surgery + radiotherapy o Surgery + chemotherapy o Surgery + radiotherapy + chemotherapy	25 (69) 11 (31) - 5 (14) - 3 (8) - 3 (8)
*All values median (IQR)*	
Months since cancer surgery	19.5 (26)
Months since last cancer treatment	17.5 (27)
*All values n (%)*	
Current pelvic floor muscle training - Less than once a month - At least once a month - At least once a week - At least three times a week	16 (44) - 1 (3) - 1 (3) - 7 (19) - 7 (19)
Previous pelvic floor muscle training	7 (19)

*IQR* inter quartile range, *TAFE* technical and further education, *PhD* Doctorate of Philosophy, *HRT* hormone replacement therapy

All data presented as *n* (%) unless otherwise stated

### Feasibility outcomes of recruitment, retention and adherence

**Fig. 2:**
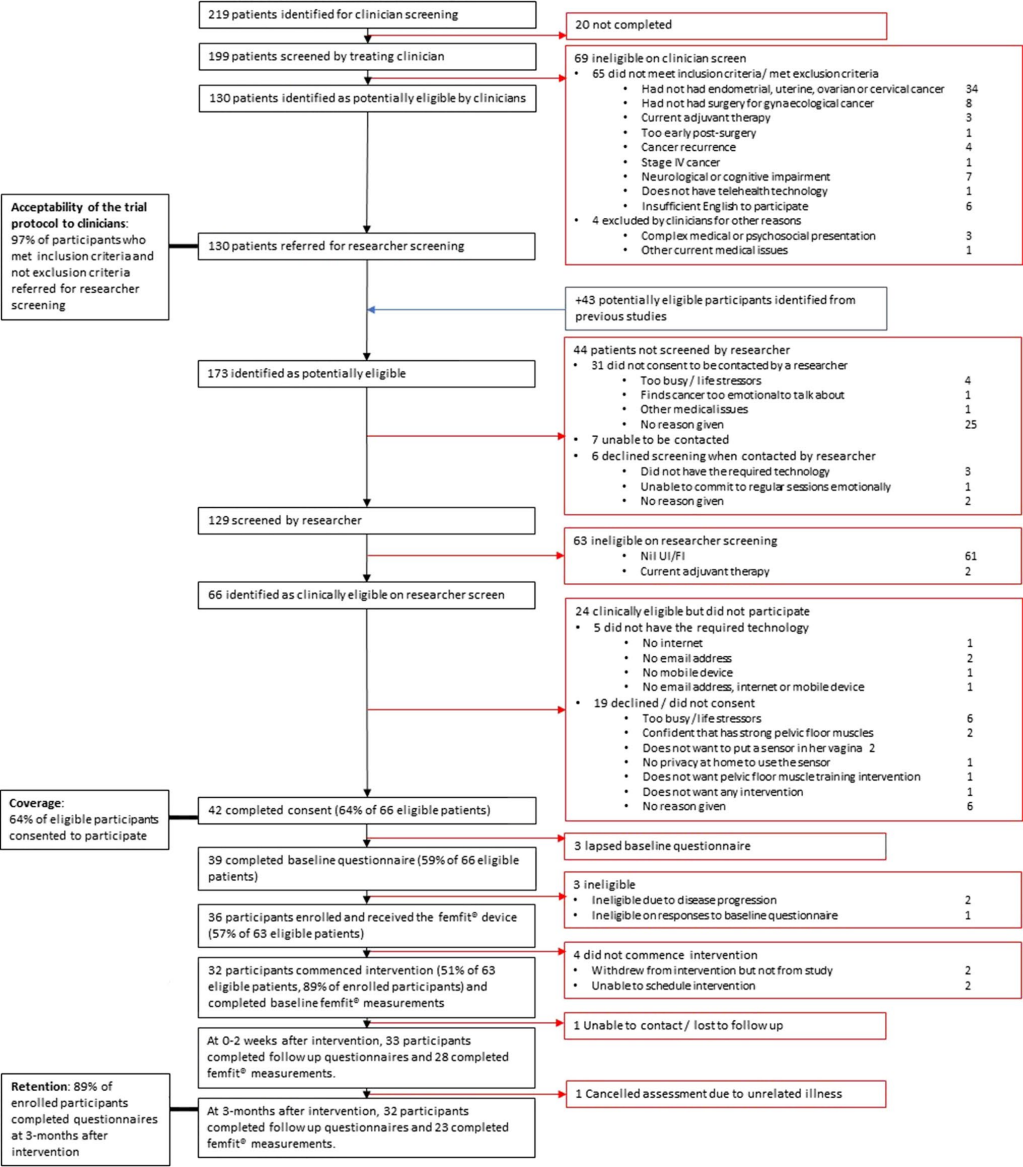
Participant flow

Figure 2 presents the participant flow through the trial. Coverage was 64%, with 42/66 of eligible patients consenting to the study, of whom three were later identified as ineligible. Reasons for ineligibility and for patients declining to participate are shown in Fig. 2. Of the 36 participants who enrolled in the study, 32 (89%) received the intervention, and 32 (89%) provided questionnaire data at the 3-month follow-up timepoint.

Of the 32 participants who received the intervention, 30 (94%) attended at least six of the seven telehealth sessions. The mean percentage of daily PFMT sessions completed was 79% (SD ± 21%). Twenty-four participants (75%) completed five-to-seven PFMT sessions per week, and only three participants (9.4%) completed fewer than three PFMT sessions per week. At the 3-month follow-up time point, 25/32 (78%) participants reported they were doing regular PFMT, with 16/32 reporting that they performed PFMT at least three times a week.

There were no major adverse events. Three participants reported one minor adverse event each throughout the duration of the study. In each instance, this was a small amount of *per vaginam* bleeding with use of the intra-vaginal sensor. Participants were advised to avoid using the sensor between the fortnightly physiotherapy sessions, to use water-based lubricant to insert the sensor, and to continue with their other gynaecology-oncology prescribed treatments of dilator therapy and topical oestrogen if these had been prescribed previously. Each of these trial-related events resolved within 24 h with no recurrence or further concerns.

### Fidelity to treatment receipt of the intervention

A total of 180 review sessions were attended by participants. Recall of the home exercise program was assessed in 177 sessions. The exercise program could be recalled in detail and/or had been completed using the femfit® application and was followed closely in 156 sessions (88%) or was followed but in a different position from the standard protocol in 14 sessions (8%). The home exercise program could not be described clearly by the participants and had not been completed according to the femfit® exercise diary in eight sessions (5%). Five participants (14%) also reported using the femfit sensor in-between telehealth sessions, rather than only in the sessions with the therapist as instructed.

### Acceptability of the intervention components

**Fig. 3:**
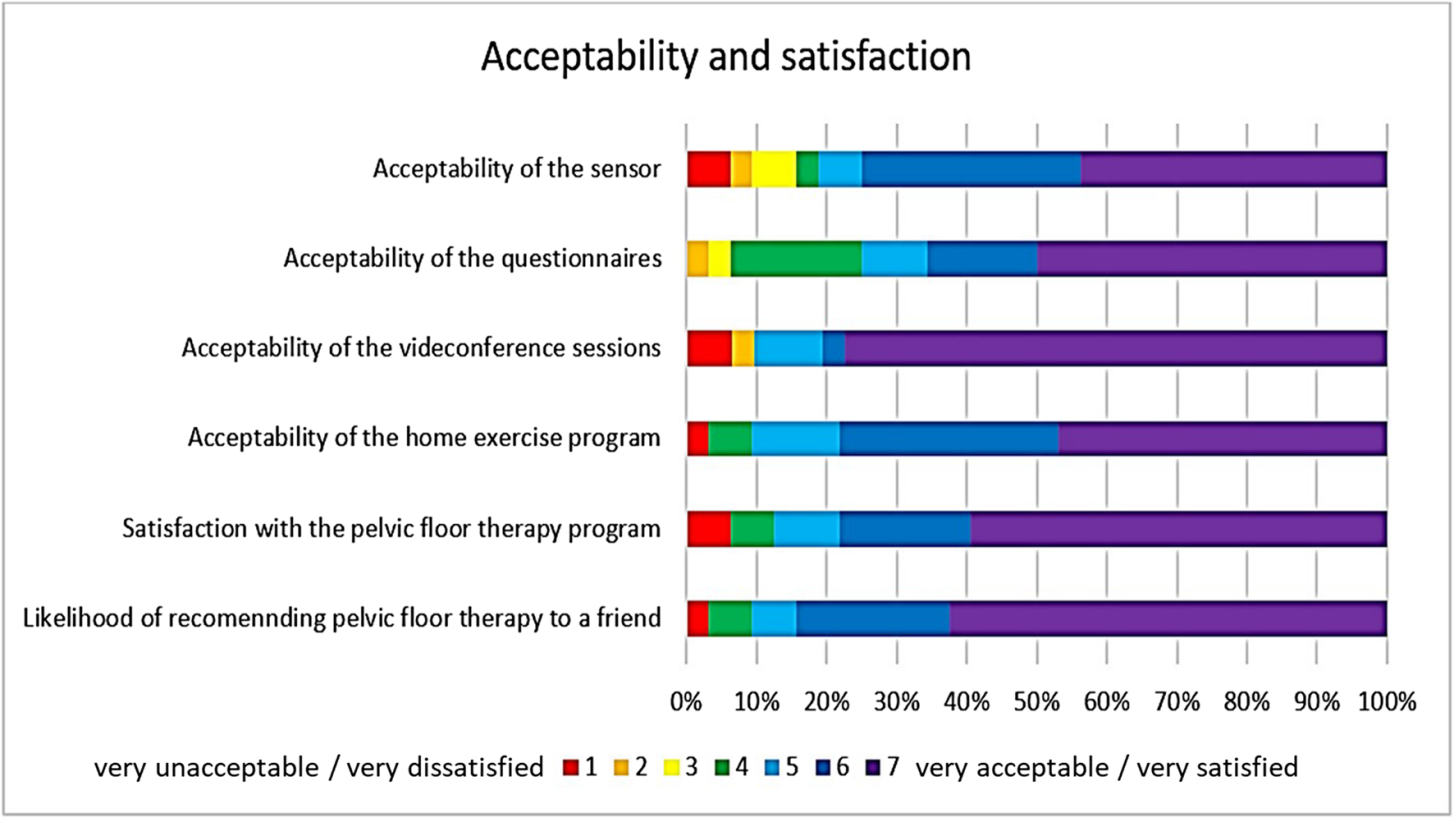
Acceptability and satisfaction

Most participants (*n*=24, 77%) rated the videoconference sessions as very acceptable, and 14 (44%) rated the intra-vaginal sensor as very acceptable. As seen in Fig. 3, the majority of participants rated all components of the program very acceptable or close to very acceptable. Nineteen participants (59%) rated their overall satisfication as very satisfied. Two participants who withdrew from the intervention prior to their first session scored the acceptability of the sensor and video conference sessions and satisfaction as very unacceptable/very dissatisfied.

### Clinical outcomes

All pelvic floor clinical outcomes improved immediately post-intervention (Table 2); however, the magnitudes of these improvements were small. The prevalence of UI and FI decreased (UI: MD −3%, 95%CI −13%, 6%; FI: MD −19% 95%CI −4%, −35%). Mean scores improved for the ICIQ-UI-SF (MD −1.65, 95%CI − 3.00, −0.15), and the ICIQ-B domains: anorectal symptoms (MD −0.58, 95%CI −1.08, −0.08); control (MD −0.62, 95%CI −1.89, 0.62); and impact (MD −1.46, 95%CI −2.85, −0.08). Pelvic floor muscle outcomes improved marginally, with PFM MVC improving by 0.16 mmHg (95%CI −5.0, 4.5) and PFM endurance by 0.42 mmHg (95%CI −1.0, 1.7). Supplementary Table 1 presents all clinical outcomes measured at all time points.

**Table 2:** Mean change in pelvic floor outcomes from baseline (time 1) to immediately post-intervention (time 2)

Outcome	Mean difference (95%CI)
Symptom prevalence (%)*	
Urinary incontinence	−3 (−13, 6)
Faecal incontinence	−19 (−4, −35)
Symptom severity and impact scores*	
ICIQ-UI-SF	−1.65 (−3.00, −0.15)
ICIQ-B anorectal symptoms	−0.58 (−1.08, −0.08)
ICIQ-B control	−0.62 (−1.89, 0.62)
ICIQ-B impact	−1.46 (−2.85, −0.08)
***Pelvic floor muscle outcomes (mmHg)***^***#***^	
PFM MVC	0.16 (−5.0, 4.5)
PFM endurance	0.42 (−1.0, 1.7)

*ICIQ-UI-SF* International Consultation on Incontinence Questionnaire Urinary Incontinence Module Short Form, *ICIQ-B* International Consultation on Incontinence Questionnaire Bowel module, *PFM* pelvic floor muscle, *mmHg* millimetres of mercury pressure, *MVC* maximal voluntary contractions

*Negative scores indicate improvement

^#^Positive scores indicate improvement

## Discussion

This study demonstrated that telehealth-delivered PFMT may be feasible to treat UI and/or FI after gynaecological cancer surgery. The high coverage, engagement, adherence, retention rates, acceptability and satisfaction support the feasibility and acceptability of this intervention to patients. The high rate of referral into the trial supports the acceptability of this intervention to gynaecology-oncology clinicians.

Our consent rate (coverage) of 64% was similar to the 63–69% found in previous similar duration studies of PFMT after gynaecological cancer that were delivered in-person or with telephone appointments [38–40]. Our retention rate for the immediate post-intervention assessment (92%) was similar to retention rates in previous studies of PFMT for UI after gynaecological cancer treatment [39]. The coverage and retention rates indicate that recruiting to a telehealth-delivered PFMT intervention is feasible compared to other modes of delivering PFMT in the gynaecology-oncology setting. Furthermore, the high engagement of our participants (94% attending ≥ 6 of 7 sessions) was comparable to previous cohort studies of PFMT in gynaecological cancer survivors that were conducted with in-person intervention [40] or in-person assessment and a home biofeedback device with telephone support [39]. The moderately high adherence rate of our participants to the daily PFMT program (mean adherence 79%) was also consistent with the 76–80% mean adherence rate reported in previous studies of PFMT conducted using home biofeedback devices in gynaecological cancer survivors [39] and breast cancer survivors [15], despite differing frequency (weekly) and modes of therapist contact (phone, videoconference plus emails) [15, 39]. These findings indicate that delivering PFMT entirely via telehealth for gynaecological cancer survivors may be a feasible alternative to other modes for delivering PFMT. It highlights that investment of research resources in RCTs to investigate the clinical efficacy of telehealth-delivered PFMT for UI and/or FI after gynaecological cancer treatment is warranted. In addition, RCTs directly comparing different modes of delivery of PFMT that with same frequency and volume of therapist contact, and motivational strategies in each arm may be needed to assess whether the mode of delivery affects engagement, adherence and clinical outcomes.

There were no major adverse events, and the three participants who experienced minor adverse events had previously undergone radiotherapy; therefore, the small amount of blood loss upon insertion of the sensor was likely related to the effect of previous radiotherapy on the vaginal tissues and was not an un-expected event. Use of this device to train PFM strengthening can therefore be considered to be safe. For blood loss of more than a small amount, not resolving within 48 h, or that recurred, medical specialist review would be recommended.

Although most participants were satisfied with the intervention, two participants (6%) were very dissatisfied and two (6%) rated satisfaction halfway between very dissatisfied and very satisfied, indicating neutral satisfaction. This contrasts with 95–100% of participants being satisfied or very satisfied in the studies by Bernard et al. [39] and Colombage et al. [15]. The participants in our study who were dissatisfied had been unable to schedule their first videoconference session and did not receive the intervention; therefore, it is unclear whether their dissatisfaction was related to not receiving the intervention, or their perception of the nature of the intervention. Of the participants who received the intervention, most were very satisfied. However, patients who considered the intervention components unacceptable would be less likely to enrol in the study and participate in the intervention, creating a selection bias, demonstrated by the two patients who declined to participate because they did not want to use an intra-vaginal sensor. Regarding technology, patients could only participate if they had a mobile device with Bluetooth and Internet access, and the capacity to download applications. This biases the participant group to patients who are willing to use such technology. While our consent rate of 6 4% of eligible patients supports the acceptability of the intervention to many patients, it is important to consider that some intervention components may be barriers for some patients and could be explored qualitatively.

The mean improvement in the ICIQ-UI SF (−1.65) did not reach the minimum important difference for this outcome measure (−2.5) [41]. Minimum clinically important differences have not been reported for the other pelvic floor clinical outcomes in this study. The mean improvement in PFM MVC (0.16 mmHg) was much smaller than that reported in previous studies of PFMT for UI in gynaecological cancer survivors (21.78 cmH_2_O=16 mmHg) [42], or breast cancer survivors (4.8 mmHg) [15]. These differences may be affected by measurement device differences [42], different methods of calculating MVC [15] and differences in participant characteristics between breast and gynaecological cancer survivors [15]. Six of our participants also reported having COVID, and six others reported non-COVID upper respiratory tract infections during the intervention period, which may have negatively affected post-intervention outcomes. A larger RCT is therefore needed to assess clinical efficacy given this cohort study. However, the mean difference and 95%CIs for the symptom severity and impact scores provide promising data, a signal that there may be a positive effect of the intervention.

### Limitations

Some limitations need noting. This study was designed to assess feasibility rather than clinical efficacy. As such, the pelvic floor clinical outcomes data must be interpreted with caution due to the single cohort design and small sample size. The validity and reliability of the femfit® have been tested in supervised settings; however, when used remotely, the therapist is unable to visually check the sensor placement. If the sensor is not inserted far enough, intra-vaginal pressure can be misrepresented as intra-abdominal pressure. Women who have treatment for gynaecological cancer may have shortened vaginal length [43], which could prevent the sensor being inserted far enough. Further studies investigating the validity of remote assessment using the femfit® device are warranted. Studies investigating hybrid models of care, in which both in-person and telehealth-delivered PFMT sessions are provided, could also be considered to address this limitation by providing digital palpation and observation to ensure correct pelvic floor muscle contraction. One option for such a model could involve pelvic floor physiotherapy assessment while patients are in the hospital or gynaecology-oncology clinic for assessment, treatment or monitoring, with physiotherapy follow-up care then provided in the community via telehealth. In addition, the PFM endurance measurements were taken during a contraction in which participants were instructed to contract ‘to halfway’, then ‘as much as you can’, then ‘reduce to half effort’. Target pressure lines that appeared on the participants’ screens during these contractions were set at 5 mmHg (halfway) and 10 mmHg (maximal), and participants who were able to generate greater pressure may have reduced their effort based on this visual feedback, preventing us from observing their maximal pressure for endurance over this time. The acceptability and satisfaction observed on our questionnaire may have been influenced by selection bias. As described above, selection bias may reduce the generalisability of the findings of the acceptability and satisfaction questionnaire. Despite these limitations, this study has shown that recruiting to and delivering telehealth-delivered PFMT for UI and/or FI in the gynaecology-oncology clinic setting are feasible and safe.

## Conclusion

This study has demonstrated the feasibility of recruiting to the telehealth-delivered PFMT intervention in the gynaecology-oncology clinic setting, and the feasibility and safety of delivering this intervention to patients who had had gynaecological cancer surgery. Our findings showed that telehealth-delivered PFMT using biofeedback may be feasible and acceptable to manage UI and/or FI after gynaecological cancer treatment. Larger RCTs are warranted to investigate clinical efficacy of telehealth-delivered PFMT for UI and/or FI after gynaecological cancer treatment.

### Supplementary information

Supplementary file 1 Supplementary table 1 Clinical outcome measures at each time point (DOCX 15 kb)

## Data Availability

The datasets generated during and/or analysed during the current study are not publicly available as these datasets could be used to identify research participants.
