# Supplementary material for: Pelvic floor muscle training delivered via telehealth to treat urinary and/or faecal incontinence after gynaecological cancer surgery: a single cohort feasibility study
Source: Support Care Cancer. 2023 Sep 23;31(10):589. doi: 10.1007/s00520-023-08050-5 (PMC10517895; doi:10.1007/s00520-023-08050-5)
Supplement: Supplementary file 1 — Supplementary table 1 Clinical outcome measures at each time point (DOCX 15 kb) [file 520_2023_8050_MOESM1_ESM.docx]

# Supplementary table 1: Clinical outcome measures at each time point

| **Outcome** | **Time 1:**  **Pre-intervention** | **Time 2:**  **Post-intervention** | **Time 3:**  **3-month follow-up** |
| --- | --- | --- | --- |
| *All values Proportion % (95%CI)* | | | |
| **Symptom prevalence** | | | |
| Urinary incontinence | 97 (91, 100) | 94 (84, 100) | 94 (84, 100) |
| Faecal incontinence | 85 (72, 97) | 67 (52, 82) | 63 (44, 78) |
| *All values Mean (95%CI)* | | | |
| **Severity and impact scores** | | | |
| ICIQ-UI-SF | 9.5 (8.4, 10.8) | 8.0 (5.7, 10.4) | 7.4 (5.5, 9.2) |
| ICIQ-B anorectal symptoms | 5.3 (4.7, 6.0) | 4.7 (3.9, 5.9) | 4.7 (4.0, 5.6) |
| ICIQ-B control | 5.6 (4.0, 7.5) | 4.8 (3.3, 6.5) | 4.8 (3.2, 6.6) |
| ICIQ-B impact | 6.4 (3.8, 9.4) | 4.9 (2.7, 7.8) | 5.8 (2.9, 9.1) |
| **Pelvic floor muscle outcomes** | | | |
| PFM MVC | 21.9(16.1, 28.1) | 21.7 (18.3, 25.8) | 21.0 (17.7, 25.2) |
| PFM endurance | 7.7 (6.2, 9.5) | 8.0 (6.8, 9.2) | 8.4 (7.1, 9.8) |
| **European Organization for Research and Treatment of Cancer Quality of Life Core Questionnaire domains** | | | |
| Physical function | 82.5 (77.1, 87.9) | 80.4 (73.8, 86.0) | 78.3 (71.5, 84.8) |
| Role function | 80.7 (73.4, 88.5) | 82.8 (74.5, 90.6) | 76.6 (65.6, 85.9) |
| Dyspnoea | 24.0 (16.7, 32.3) | 16.7 (9.4, 26.0) | 18.8 (12.5, 26.0) |
| Insomnia | 33.3 (22.9, 45.8) | 33.3 (24.0, 42.7) | 26.0 (15.6, 36.5) |
| Appetite loss | 11.5 (6.3, 18.7) | 13.5 (6.3, 21.9) | 11.5 (4.2, 19.8) |
| Nausea | 6.3 (2.6, 10.9) | 7.3 (2.1, 14.1) | 3.1 (0.0, 8.3) |
| Constipation | 8.3 (4.2, 13.5) | 10.4 (4.2. 16.7) | 8.3 (2.1, 15.6) |
| Diarrhoea | 15.6 (6.3, 27.1) | 14.6 (6.3, 24.0) | 10.4 (3.1, 19.8) |
| Fatigue | 37.2 (30.9, 44.1) | 34.0 (26.0, 41.7) | 33.0 (25.7, 40.6) |
| Pain | 28.7 (18.7, 39.1) | 38.5 (27.6, 41.7) | 29.2 (19.8, 41.7) |
| Emotional function | 78.7 (71.1, 85.7) | 81.0 (74.2, 87.5) | 76.0 (69.3, 82.8) |
| Cognitive function | 82.8 (77.6, 88.0) | 82.3 (75.5, 89.6) | 86.5 (80.7, 91.7) |
| Social function | 82.3 (72.9, 90.6) | 69.8 (57.3, 82.3) | 78.7 (66.7, 88.5) |
| Financial distress | 15.6 (7.3, 25.0) | 13.5 (5.2, 21.9) | 8.3 (3.1, 13.5) |
| General health status / QoL | 62.2 (55.7, 68.2) | 65.1 (59.6, 70.8) | 61.7 (52.9, 69.8) |
| Abbreviations: ICIQ-UI-SF International consultation on Incontinence Questionnaire Urinary Incontinence module Short Form; ICIQ-B International Consultation on Incontinence Questionnaire Bowel module; PFM pelvic floor muscle; MVC maximal voluntary contractions; QoL Quality of Life | | | |
